# Evaluation of 16S rRNA Gene Primer Pairs for Monitoring Microbial Community Structures Showed High Reproducibility within and Low Comparability between Datasets Generated with Multiple Archaeal and Bacterial Primer Pairs

**DOI:** 10.3389/fmicb.2016.01297

**Published:** 2016-08-23

**Authors:** Martin A. Fischer, Simon Güllert, Sven C. Neulinger, Wolfgang R. Streit, Ruth A. Schmitz

**Affiliations:** ^1^Department of Biology, Institute for General Microbiology, Christian-Albrechts-Universität zu KielKiel, Germany; ^2^Biozentrum Klein Flottbek, Institute of Microbiology & Biotechnology, Universität HamburgHamburg, Germany; ^3^omics2view.consulting GbRKiel, Germany

**Keywords:** amplicon sequencing, next-generation sequencing, archaea, metagenome, 16S microbial community, microbial communities

## Abstract

The application of next-generation sequencing technology in microbial community analysis increased our knowledge and understanding of the complexity and diversity of a variety of ecosystems. In contrast to Bacteria, the archaeal domain was often not particularly addressed in the analysis of microbial communities. Consequently, established primers specifically amplifying the archaeal 16S ribosomal gene region are scarce compared to the variety of primers targeting bacterial sequences. In this study, we aimed to validate archaeal primers suitable for high throughput next generation sequencing. Three archaeal 16S primer pairs as well as two bacterial and one general microbial 16S primer pairs were comprehensively tested by *in-silico* evaluation and performing an experimental analysis of a complex microbial community of a biogas reactor. The results obtained clearly demonstrate that comparability of community profiles established using different primer pairs is difficult. 16S rRNA gene data derived from a shotgun metagenome of the same reactor sample added an additional perspective on the community structure. Furthermore, *in-silico* evaluation of primers, especially those for amplification of archaeal 16S rRNA gene regions, does not necessarily reflect the results obtained in experimental approaches. In the latter, archaeal primer pair ArchV34 showed the highest similarity to the archaeal community structure compared to observed by the metagenomic approach and thus appears to be the appropriate for analyzing archaeal communities in biogas reactors. However, a disadvantage of this primer pair was its low specificity for the archaeal domain in the experimental application leading to high amounts of bacterial sequences within the dataset. Overall our results indicate a rather limited comparability between community structures investigated and determined using different primer pairs as well as between metagenome and 16S rRNA gene amplicon based community structure analysis. This finding, previously shown for Bacteria, was as well observed for the archaeal domain.

## Introduction

The investigation of the microbial community composition allows a detailed insight in diversity and potential ecosystem function and fosters understanding of complex microbial processes (Vanwonterghem et al., 2014). Recent years have seen a strong increase in sequencing approaches targeting microbial communities via amplicon sequencing or metagenomic and metatranscriptomic approaches (Turnbaugh et al., 2007; Hamady et al., 2008; Raes and Bork, 2008; Caporaso et al., 2012; Grosskopf and Soyer, 2014; Ininbergs et al., 2015). These approaches play an important role in monitoring and comparing large numbers of samples in terms of their microbial composition (Caporaso et al., 2012; Kozich et al., 2013; Sundberg et al., 2013). The by far most often used marker for prokaryotic diversity studies is the 16S rRNA or its corresponding gene. The first to perform extensive research based on the 16S region were Woese and Fox (Woese and Fox, 1977; Woese et al., 1990). Their profound and passionate work led to the discovery of the third domain of life, the Archaea (Woese et al., 1990). Since then, the contribution of the archaeal domain to ecosystem function and diversity was often underestimated in many research fields and studies. While the bacterial fraction of many environments was extensively studied, the Archaea were often not specifically addressed. This underestimation of archaeal contribution to biology can be observed in a variety of studies from Sanger sequencing-based approaches to 454- and MiSeq-based high-throughput sequencing based studies (Frank et al., 2007; Herlemann et al., 2011; Ding and Schloss, 2014; Wang et al., 2015). Many reports focusing on Archaea appear to explore extreme environments like hot springs (Beam et al., 2015), deep sea volcanos (Reysenbach et al., 2006), and black smokers (Takai and Nakamura, 2011) to only mention a few, which further promotes the image of Archaea to represent extremophiles. On the contrary, Archaea are ubiquitously found under rather mesophilic conditions like in fresh and marine waters (DeLong, 1992; DeLong et al., 1994; Karner et al., 2001; Stahl and de la Torre, 2012), biogas reactors (Sundberg et al., 2013), and soil (Leininger et al., 2006), the intestinal tract of termites (Paul et al., 2012), ruminants (Jeyanathan et al., 2011; Kittelmann et al., 2013), but also on the human skin (Probst et al., 2013; Oh et al., 2014), or in the intestine (recently reviewed in Bang and Schmitz, 2015), where they complete the microbiome together with their bacterial, eukaryotic and viral partners.

Regarding biogeochemical cycles, the archaea harbor the unique trait of the methanogenic pathway (Offre et al., 2013). The methane emission by archaeal activity is used in industrial scale as a beneficial source of renewable energy in biogas reactors but is problematic when observed under the perspective of greenhouse gas emission. Two major sources of anthropogenic methane emission is livestock and rice patty fields (Yusuf et al., 2012), two habitats known to harbor methanogenic archaeal communities (Janssen and Kirs, 2008; Kittelmann et al., 2013; Breidenbach and Conrad, 2014) and both contributing notable amounts to the overall anthropogenic greenhouse gas emission (Wuebbles, 2002; Ripple et al., 2014). Additionally, in livestock methane production by the enteric community leads to an energy loss for the host by the emission of the energy rich methane and several studies investigate potential inhibitors of archaeal methane production (Goel and Makkar, 2012; Duin et al., 2016). The archaeal communities of ruminants has therefore been in the focus of several studies in recent years (Skillman et al., 2004; Jeyanathan et al., 2011; Kim et al., 2011; Singh et al., 2012; Tymensen and McAllister, 2012; Kittelmann et al., 2013; Henderson et al., 2015), some of them involving primer evaluation for the archaeal community (Watanabe et al., 2004; Gantner et al., 2011) or extending the microbiome research by adding results for protozoa and fungi (Kittelmann et al., 2013).

In an extensive study, Klindworth et al. (2013) performed a detailed *in-silico* evaluation of a 16S rRNA primer dataset containing 175 primers and 512 primer pairs, with 72 primers targeting archaeal 16S gene sequences. Primers and primer pairs were tested against the SILVA 16S non-redundant reference database to estimate their accuracy and phylogenetic coverage. Inspired by this study, we tested the experimental applicability of several primer combinations—some recommended in the above mentioned study, others supplemented based on literature review. After initial *in-silico* validation, the six most promising primer pairs were chosen; three targeting the archaeal, two the bacterial and one overall prokaryotic 16S rRNA gene sequence. These primer pairs showed high *in-silico* coverage and specificity, and were used to investigate the microbial community of an anaerobic, mesophilic biogas reactor, a habitat known to host a diverse community of Archaea and Bacteria (Eikmeyer et al., 2013; Sundberg et al., 2013). To eliminate disruptive effects and ensure maximum comparability, we used the same template DNA extracted from one sample of the above mentioned biogas reactor for all approaches. Shotgun metagenomic approaches have been introduced into community analysis (Venter et al., 2004) and bear the additional advantage of hinting toward ecosystem potential beside the taxonomic information (Vanwonterghem et al., 2014). Renunciation of 16S rRNA gene amplification is another positive effect of shotgun metagenomics, as it rids the data of primer bias (Shakya et al., 2013; Logares et al., 2014; Tremblay et al., 2015). Thus, as an additional and independent approach, we used 16S rRNA gene data obtained in a very comprehensive metagenome sequencing approach of the same biogas fermenter material (Güllert et al., 2016) as a reference point for comparison.

This study aims to estimate the effect of primer choice on the observed sequence composition of a diverse microbial community. Contrary to other studies focusing on the evaluation of bacterial 16S rRNA primers, we focus here on the evaluation and observation of the archaeal community in more detail. We further critically discuss the reliability of *in-silico* primer evaluation in terms of unspecific amplification and target specificity in application to environmental samples. Additionally, the 16S rRNA gene amplicon based community profiles were compared to the 16S rRNA gene sequences extracted and assembled from shotgun metagenomic data.

## Materials and methods

### Anaerobic sludge sample

For nucleic acid extraction, one sample was taken from a mesophilic (40°C) full-scale biogas plant (power output, 540–580 kWh) located near Cologne (Germany) on May 27th 2013. Main substrate for anaerobic digestion was maize silage (69%), cattle manure (19%), and dry poultry manure (12%). The pH of the reactor was 7.96, volatile fatty acids 3.06 g acetic acid equivalents/L, total inorganic carbon 17.7 g CaCO_3_/L, free ammonia 2.98 g/L. One liter of sample material was taken under standardized conditions and kept at 4°C during transport to the laboratory, where it was stored at −20°C until DNA extraction.

### DNA extraction

Two milliliter of frozen sample were homogenized prior to extraction using a Dismembrator-U mechanical mortar (Sartorius Stedim Biotech GmbH, Göttingen, Germany) for 5 min at 2.500 rpm. DNA was extracted from the homogenate with the CTAB (Cetrimonium bromide) -chloroform:isoamyl alcohol based protocol as described by Weiland et al. (2010). Due to high concentration of humic acids in the final DNA extracts, DNA was further purified by the FastDNA™ SPIN Kit for Soil (MP Biomedicals, Solon, UH, USA). After extraction, purity was checked spectrophotometrically using the NanoDrop 1000 Fluorospectrometer (Thermo Fisher Scientific, Bremen, Germany) by measuring the absorbance at 260 and 280 nm (260/280 = 1.57).

### 16S primer selection and *in-silico* evaluation

Three different primer pairs targeting the archaeal 16S rRNA gene region were selected from recent publications (Takai and Horikoshi, 2000; Baker et al., 2003; Yu et al., 2005; Frank et al., 2007; Fierer et al., 2008; Park et al., 2008; Morales and Holben, 2009; Claesson et al., 2010; Herlemann et al., 2011; Klindworth et al., 2013). The main criteria for their selection were (i) *in-silico* specify for archaea, (ii) low bias in amplifying specific groups of prokaryotes and (iii) amplicon length between 250 and 600 base pairs (bp), suited for next-generation sequencing techniques such as 454 Pyrosequencing or Illumina MiSeq. Two primer pairs targeting bacterial 16S rRNA gene (Lane et al., 1985; Hamady and Knight, 2009; Herlemann et al., 2011) and one primer pair for both bacterial and archaeal 16S rRNA gene (Klindworth et al., 2013) were selected applying the same criteria.

*In-silico* evaluation of the selected primers was performed using the arb-SILVA online tools TestPrime and TestProbe (Klindworth et al., 2013) employing the non-redundant SILVA 16S small subunit reference database (ssu r123, SILVA Ref NR; Pruesse et al., 2007). The results for the tested primers and primer pairs are listed in Tables 1, 2.

**Table 1:** *****In-silico*** evaluation results for selected primers**.

**Primer name**	**Primer sequence 5′ to 3′**	**Reference**	**T_M_**	**Coverage of domain**		
				**Archaea**	**Bacteria**	**Eukarya**
S-D-Arch-0787-a-S-20	ATTAGATACCCSBGTAGTCC	Yu et al., 2005	56.6	88.3	7.7	0
S-D-Arch-0787-a-A-20	GGACTACVSGGGTATCTAAT					
S-D-Arch-1043-a-A-16	GCCATGCACCWCCTCT	Yu et al., 2005	54.3	82.8	0	0
S-D-Arch-0519-a-S-15	CAGCMGCCGCGGTAA	Park et al., 2008	54.7	95.7	94.4	92.4
S-D-Arch-1041-a-A-18	GGCCATGCACCWCCTCTC	Baker et al., 2003	60.5	81.9	0	0
S-D-Arch-0349-a-S-17	GYGCASCAGKCGMGAAW	Takai and Horikoshi, 2000	56.4	81	0	0
S-D-Bact-0785-a-A-21	TACNVGGGTATCTAATCC	Claesson et al., 2010	51.8	92.1	92.3	0.8
S-D-Bact-0007-a-S-20	AGAGTTTGATCCTGGCTCAG	Frank et al., 2007	57.3	0	65.9	0
S-D-Bact-0338-a-A-19	TGCTGCCTCCCGTAGGAGT	Fierer et al., 2008	61.0	0	90.6	0
S-D-Bact-0341-b-S-17	CCTACGGGNGGCWGCAG	Herlemann et al., 2011	61.2	0.6	94.4	0
S-D-Bact-0907-a-A-20	CCGTCAATTCMTTTRAGTTT	Morales and Holben, 2009	51.2	0.8	90.2	75.4

*The T_M_ temperature was calculated using the NEB online tool. The Coverage was estimated using the SILVA TestProbe tool with the sSU-NR database version 123 and refers to the percentage of potentially covered taxa in the different domains allowing non and one mismatch (MM) during primer annealing*.

**Table 2:** **Results of the ***in-silico*** evaluation of the selected primer pairs**.

**Primer pair**	**Pair name**	**T_A(exp)_**	**Reference**	**fragment length (nt)**	**Coverage of domain no MM**			**Coverage of domain 1 MM**		
					**Archaea**	**Bacteria**	**Eukarya**	**Archaea**	**Bacteria**	**Eukarya**
S-D-Arch-0787-a-S-20/S-D-Arch-1043-a-A-16	ArchV56	60	Yu et al., 2005	272	76.50	0.00	0.00	84	0	0
S-D-Arch-0519-a-S-15/S-D-Arch-1041-a-A-18	ArchV46	60	Ogawa et al., 2014	540	79.00	0.00	0.00	93.2	0	0
S-D-Arch-0349-a-S-17/S-D-Arch-0786-a-A-20	ArchV34	56	This study	457	74.00	0.00	0.00	90.7	0	0
S-D-Arch-0519-a-S-15/S-D-Bact-0785-a-A-21	PrkV4	60	van Bleijswijk et al., 2015	287	88.60	88.20	0.70	95.2	95	1.4
S-D-Bact-0007-a-S-20/S-D-Bact-0338-a-A-19	BacV12	56	Langfeldt et al., 2014	350	0.00	60.20	0.00	0	85.8	0
S-D-Bact-0341-b-S-17/S-D Bact-0907-a-A-20	BacV35	60	Güllert et al., 2016	586	0.00	85.60	0.00	60.8	95.3	0.2

*The T_A_ temperature was applied in the amplification reaction. The Coverage of the primer pairs was estimated using the SILVA TestPrime tool with the sSU-NR database version 123 and refers to the percentage of potentially covered taxa in the different domains allowing no and one mismatch (MM) during primer annealing*.

### PCR and library preparation

Primers of each tested primer pair included the 454 Adapter set A, an unique identifier barcode sequence and a linker sequence as previously described (Langfeldt et al., 2014). For each primer pair, five separate PCR preparations with 5 individual Identifiers were performed for the investigation of experimental reproducibility. Extracted DNA was adjusted to a concentration of 20 ng/μl and used as template in the amplification reactions. The PCR reaction mixtures consisted of 17.25 μl H_2_O (Carl Roth, Karlsruhe, Germany), 250 nM of the forward and reverse Primer (MWG, Ebersberg, Germany), 5 μl 5x Phusion Reaction buffer HF, 250 nM dNTP mix, 0.5 U Phusion HF DNA Polymerase (Thermo Fisher Scientific, Bremen, Germany) and 40 ng template. The annealing temperature for each primer pair was pre-calculated using the Tm Calculator online tool from NEB [version 1.8.1; New England Biolabs, Ipswitch (MA), USA (http://tmcalculator.neb.com)]. Cycling conditions for PCR amplification started with an initial denaturation step for 30 s at 95°C, followed by 30 cycles of 10 s at 95°C, 45 s at the respective annealing temperature and 30 s at 72°C and a final extension for 10 min at 72°C. All reactions were performed in triplicates with one corresponding negative control. Negative control contained no template DNA in the PCR reaction mixture and showed no amplification. Amplicons were checked for correct length and purified via agarose gel electrophoresis using the MinElute Gel Extraction Kit (Qiagen, Hilden, Germany) as previously described in Langfeldt et al. (2014). DNA concentration in eluates ranged from 7.9 to 77.5 ng/μl, as determined with a Nanodrop ND 1000 spectrophotometer (Thermo Fisher Scientific, Bremen, Germany). Samples were combined in the sequencing pool in equal concentrations. Sequencing was performed by MWG in accordance to manufacturer's recommendations on a Roche 454 GS-FLX++ system using the Titanium sequencing chemistry in two sequencing runs.

### Amplicon sequence processing

Sequences were processed using Mothur v1.35.1 (Schloss et al., 2009) as described in Weiland-Bräuer et al. (2015) with the following modifications. Since some primers contained degenerated positions, sequences containing up to five differences in the primer region and one difference in the barcode region were kept in the dataset. Only those sequences with an average Phred score (Ewing et al., 1998; Ewing and Green, 1998) ≥25 and a maximum of eight homopolymers were retained. Each primer dataset was analyzed separately. Sequence alignment was performed against SILVA-based bacterial and archaeal reference alignments (Release 102; Pruesse et al., 2007). The alignment procedure, the filtering step, the removal of chimeric sequences, and the taxonomic classification were performed as against the mothur formatted SILVA database version 123 (Pruesse et al., 2007). For further processing and comparison between samples, only the domains of interest (Archaea, Bacteria or both) were kept in the dataset used for comparison of the diversity. Unclassified sequences or sequences from a domain not targeted by the respective primer pair were removed. The classification step produced a tax.summary file, listing the abundances of the classified taxa in each sample for each primer pair. For comparison between the different primer pairs, the tax.summary files of the different primer pairs were merged using the command merge.taxsummary.

Each dataset further yielded a shared file listing the sample-by-operational taxonomic unit (OTU) distribution for a given primer pair. These files were used in the calculation of α-diversity indices on the 97 and 99% similarity level.

### Metagenome sequencing, assembly, and annotation

Sequences corresponding to the biogas reactor sample discussed here were extracted from metagenomic data (Güllert et al., 2016) available under the accession PRJNA301928 (http://www.ncbi.nlm.nih.gov/bioproject/PRJNA301928). The extracted reads were trimmed using the Trimmomatic v0.30 software (Bolger et al., 2014). For the extraction of 16S rRNA gene sequences, the software reago v1.1 (Yuan et al., 2015) was used. 336,424 reads were identified as 16S rRNA gene sequences and assembled using the Spades assembler (Bankevich et al., 2012) generating 286 contigs with a length > 200 bp and a coverage above 2. N_50_ of the generated contigs was 336 bp. Before further analysis, the coverage and length information were used to normalize the contig abundances. Taxonomic annotation of metagenomic 16S rRNA gene sequences was performed with Mothur (Schloss et al., 2009). Sequences containing ambiguous bases and/or sequences shorter than 250 bp were removed. The remaining 198 unique contigs were taxonomically classified against the SILVA database (version 123; Pruesse et al., 2007).

Amplicon sequences are accessible via ncbi (http://www.ncbi.nlm.nih.gov/bioproject/PRJNA315559) under PRJNA315559.

All following computational analysis was performed in R v3.2.1 (R Core Team, 2015) using the vegan package version 2.3-5 (Oksanen et al., 2015).

For the calculation of the alpha diversity, OTU's generated by the mothur pipeline at 97 and 99% similarity level after removal of singletons were used. OTU counts were transformed to relative abundances per sample and OTUs with abundance below 0.2% in the corresponding primer dataset were dismissed. Shannon diversity index (H) was calculated using equation 1 from filtered relative OTU counts using vegan's function *diversity()*.Shannon numbers equivalent (^1^D) was calculated using equation (2) based on the Shannon diversity which was set as exponent to base *e* to yield ^1^D (Legendre and Legendre, 1998).

(1) $$\begin{array}{l} H=-\sum_{i=1}^{N}p_{i}\ln(p_{i}) \end{array}$$

(2) $$\begin{array}{l} {}^{1}D=e^{\left(-\sum_{i\;=\;1}^{N}\;p_{i}\ln\left(p_{i}\right)\right)}=e^{H} \end{array}$$

where *p*_i_ = *n*_i_*/N* is the relative and *n*_i_ is the absolute abundance of species *i* and *N* is the total number of species within the dataset.

Beta-diversity was calculated separately for the bacterial and archaeal domain based on the merged tax.summary files listing abundances of taxonomic bins for each sample. This abundance-per-sample table was transformed and normalized using the Hellinger equation shown in Equation (3) (Legendre and Gallagher, 2001). The Hellinger transformed data was used to calculate a Bray-Curtis dissimilarity matrix by the Equation (4) (Legendre and Legendre, 1998) as well as for the redundancy analysis (RDA).

(3) $$\begin{array}{l} y_{ij}^{\prime }=\sqrt{\frac{y_{ij}}{y_{j+}}} \end{array}$$

Where *y*_ij_ is the abundance of species *i* at site *j* and *y*_*j*+_ is the sum of all species at site *j*.

(4) $$\begin{array}{l} D_{BrayCurtis}\left(i,j\right)=1-\frac{\sum_{k\;=\;0}^{n}|y_{ik}-y_{jk}|}{\sum_{k\;=\;0}^{n}\left(y_{ik}+y_{jk}\right)} \end{array}$$

where the dissimilarity (*D*_*BrayCurtis*_) between the community of two sites (*i,j*), *k* as index of the numbers of species and *y*_ik_ the abundance of species *i* at site *k*.

## Results

### Primers show high *in-silico* coverage

Primers for the amplification of the archaeal and bacterial 16S rRNA genes were selected in a way to avoid amplification of sequences outside the designated domain. The primers designated to the amplification of archaeal and bacterial 16S rRNA gene exhibited high *in-silico* coverage for both domains (between 66 and 96%). All evaluated primers for the archaeal domain covered ≥81% of all phyla in the SILVA database present in January 2016 (ssu r123, SILVA Ref NR). Bacterial primers showed an even higher theoretical coverage of over 90% (see Table 1). An exception to this was the primer S-D-Bact-0007-a-S-20 which only matched 66% of the bacterial phyla in the database. The primers designed for the combined amplification of archaeal and bacterial 16S rRNA gene sequences showed the overall highest theoretical coverage targeting 95.7% of all archaeal and 92.3% of all bacterial sequences. When one mismatch in the base pairing of the primers was allowed, the coverage of the primers designed for the amplification of archaeal 16S rRNA gene sequences increased to a coverage of ≥87% of all phyla in the SILVA. A similar increase to ≥95% of all bacterial phyla in the SILVA database was observed for the primers targeting the bacterial 16S rRNA gene sequences. A hint for possible false amplification indicated by theoretical coverage outside the designated domain was observed for the primers S-D-Arch-0787-a-S/A-20, S-D-Bact-0341-b-S-17 and S-D-Bact-0907-a-A-20. The primers S-D-Bact-0907-a-A-20, S-D-Bact-0785-a-A-21, and S-D-Arch-0519-a-S-15 showed potential coverage of the eukaryotic domain (0.8–92.4%). Considering one mismatch during the annealing progress, the potential for false amplification outside the targeted domain increased in the above mentioned primers and was additionally observed in the primers S-D-Arch-0349-a-S-17 and S-D-Bact-0338-a-A-19 in minor amounts (Table 1).

Primers were combined into pairs as listed in Table 2. *In-silico* evaluation suggested pairs ArchV46 and BacV35 to be the most promising pairs for amplification of archaeal and bacterial 16S rRNA gene sequences, respectively. The primer pairs chosen for amplification of prokaryotic 16S rRNA gene sequences covered 88.6% of the archaeal and 88.2% of the bacterial phyla in the database *in-silico*.

### High read reduction during processing in archaeal 16S rRNA gene sequences was caused by unspecific amplification

**Figure 1:**
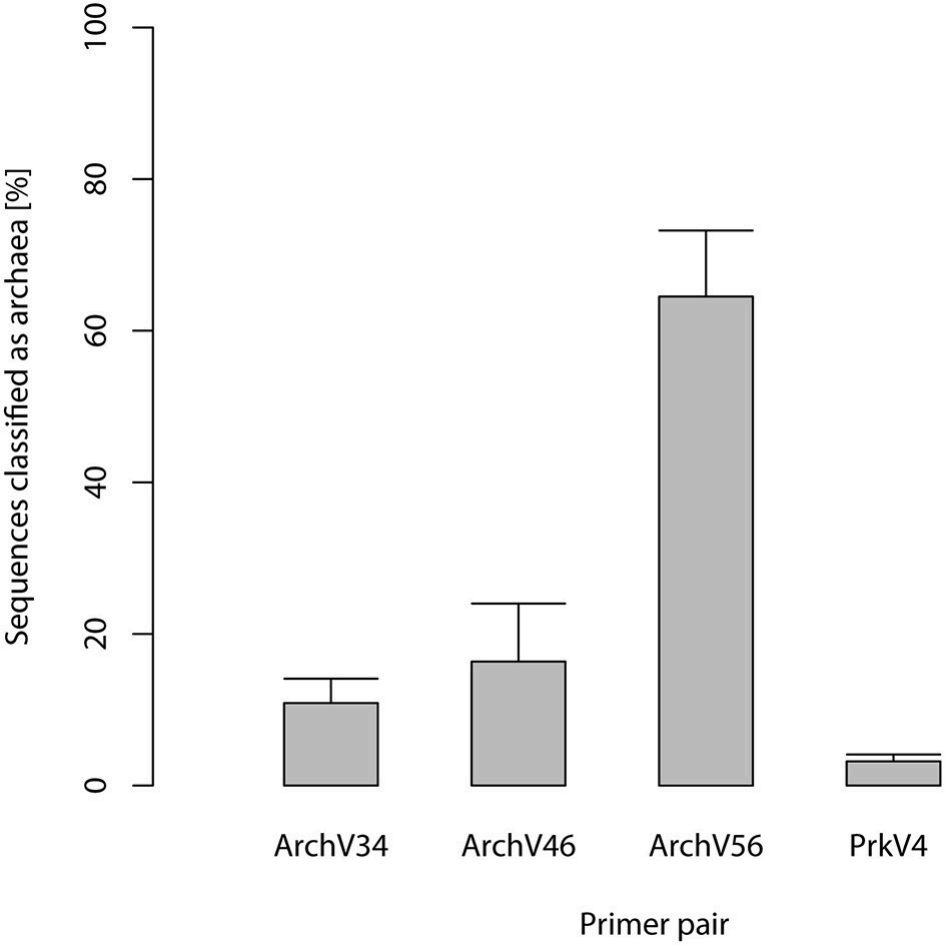
**Percentage of sequences classified as archaeal in the datasets generated applying the primers targeting the archaeal 16S ribosomal RNA gene region**. 100% refers to all sequences in the separate datasets while the percentage of reads classified as Archaea is indicated by the boxes, standard deviation between the *n* = 5 replicates is indicated by the bars.

**Figure 2:**
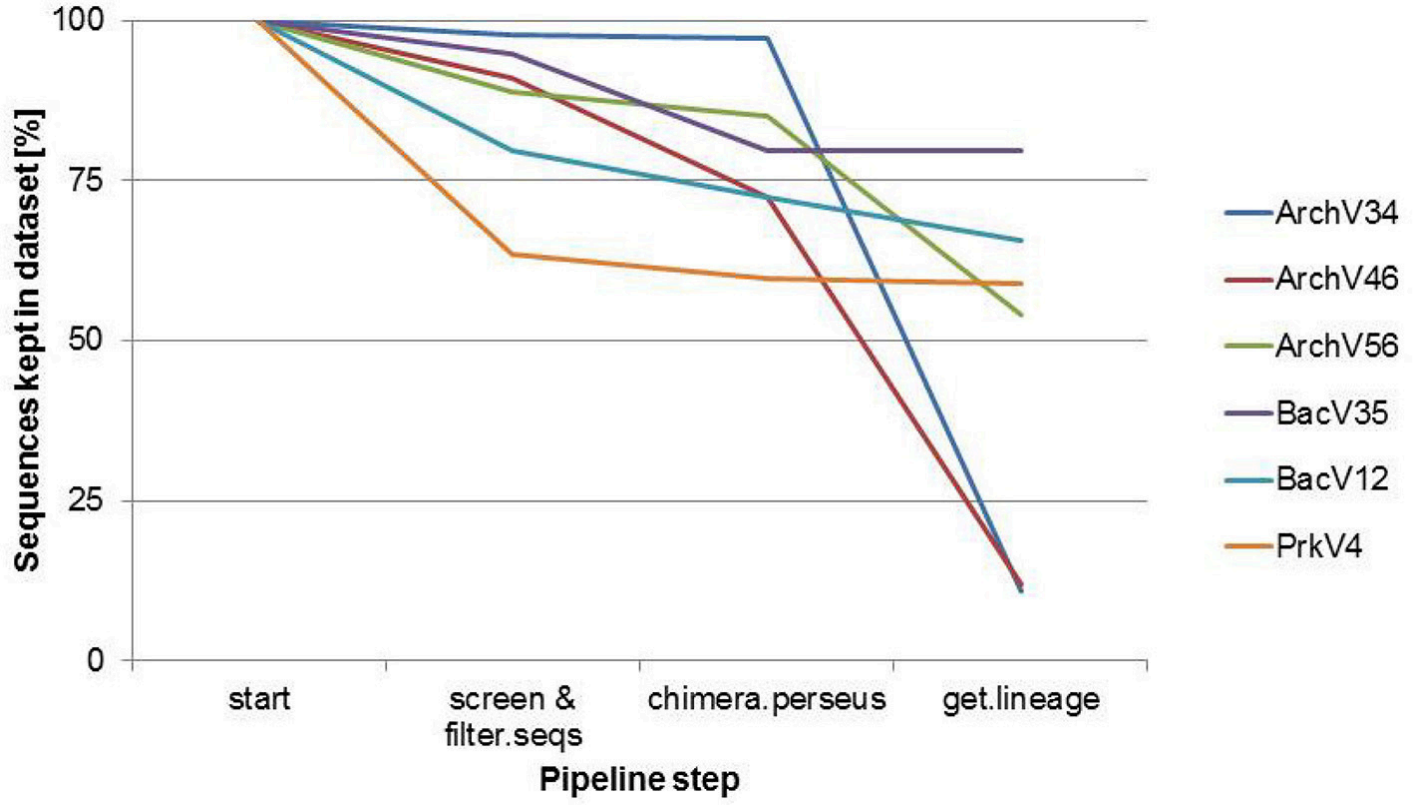
**Sequence reduction for the compared 16S rRNA gene primer pairs during the different steps of the mothur analysis pipeline**. 100% refers to the number of all sequences in each dataset at the beginning of the Mothur sequence annotation pipeline.

Selected primers pairs designed for the archaeal domain exhibited low specificity in practical application. Figure 1 shows amplicon read classification before the taxonomy-based filter step. The percentage of amplicon sequences classified as archaeal after the alignment and annotation step ranged between 64.5 and 10.89% in case of the primers designed for the detection of archaea. In the datasets of the primers designed for the amplification of bacterial 16S rRNA gene over 99% of the amplicon sequences were classified as bacterial (data not shown). The ratio of archaeal to bacterial sequences in the dataset generated with the prokaryotic primers was 3.2–96.8% (see Figure 1). Non-archaeal sequences in the archaeal datasets originated mostly from bacterial 16S rRNA gene regions, indicating unspecific amplification by the tested archaeal primer pairs. Read reduction within the mothur pipeline is visualized for each primer pair in Figure 2 and summarized in Table S1. In the primer pairs ArchV46 and ArchV34, a strong reduction of the number of sequences in the datasets was observed when filtering for the targeted domain (*get.lineague* command). The primer pairs targeting the bacterial 16S rRNA gene region as well as the PrkV4 and ArchV56 primer pair showed only minor reduction in this filtering step. Reduction in this particular processing step hints toward the deletion of unclassified sequences as well as sequences from untargeted domains.

### Taxonomic composition differs depending on used primer pair

**Figure 3:**
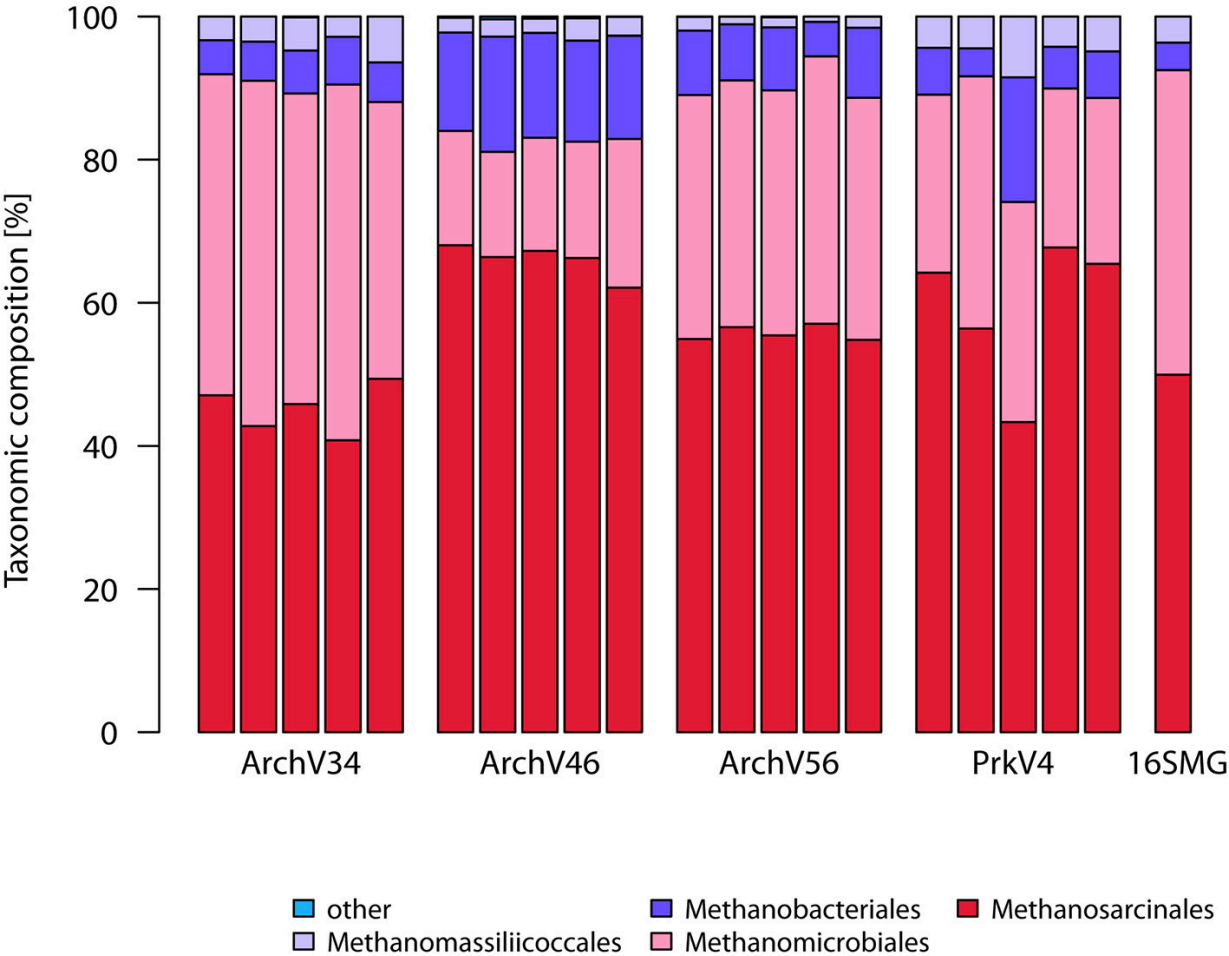
**Sequence compositions of the communities observed by the different archaeal and prokaryotic primer pairs on the order level**. Shown are the five replicates of each primer pair as well as the 16S rRNA gene sequences extracted from the metagenome (16S-MG). Annotation was done using the SILVA v123 database. Taxa with abundance below 1% were grouped as “other”.

**Figure 4:**
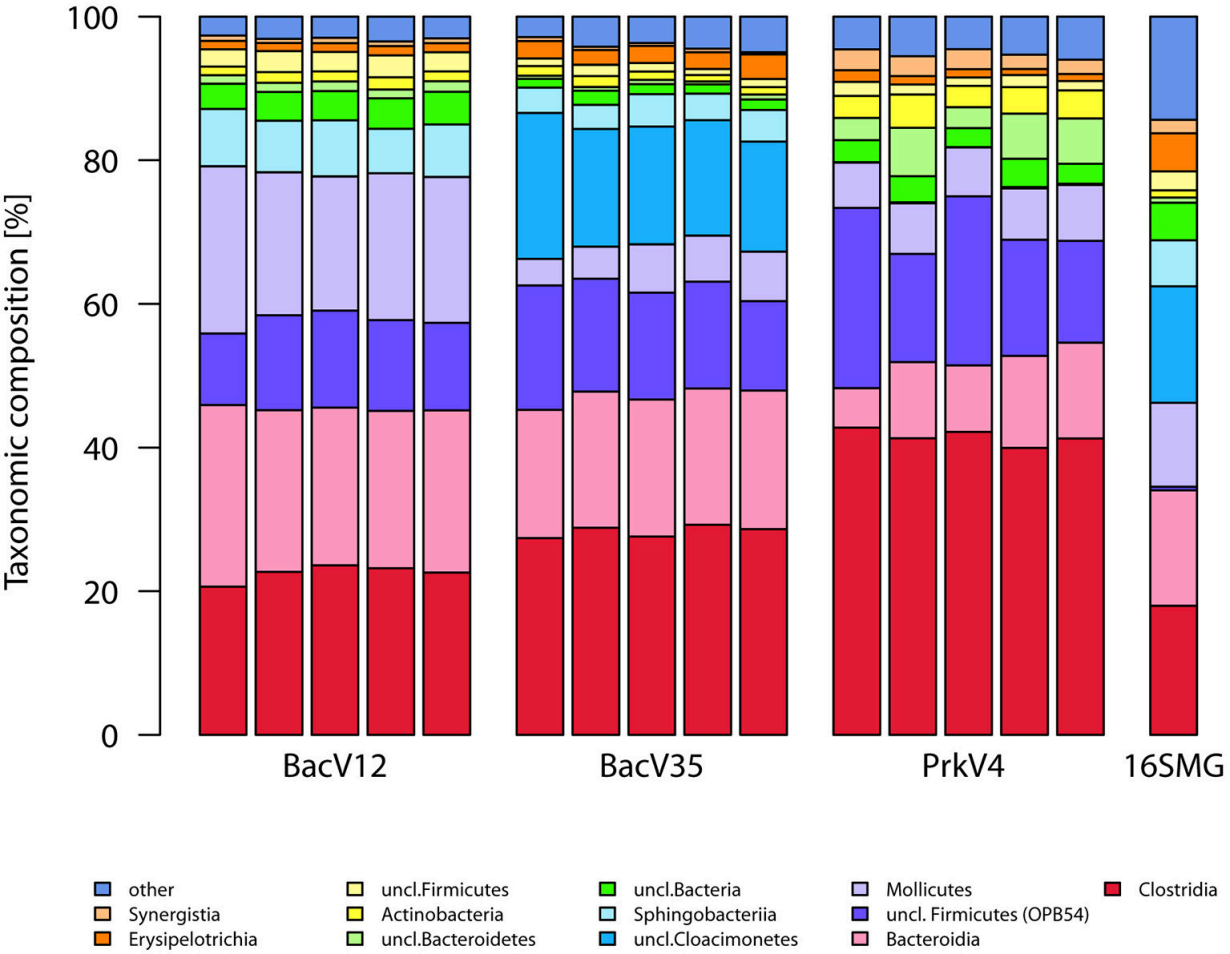
**Sequence compositions of the communities observed by the different bacterial and prokaryotic primer pairs on the class level**. Shown are the five replicates of each primer pair as well as the 16S rRNA gene sequences extracted from the metagenome data pair (16S-MG). Annotation was done using the SILVA v123 database. Taxa with abundance below 1% were grouped as “other”.

The taxonomic annotation of the sequence in the datasets generated by the tested primer pairs and extracted from the metagenome (16S rRNA gene fraction) is shown in the Figure 3 for the archaeal part on the order level as well as in Figure 4 for the bacterial part on the class level.

In the archaeal domain, all primer pairs identified sequences annotated as *Methanosarcinales* as the most dominant order (45–66% of the sequences in the datasets) followed by *Methanomicrobiales* reaching between 17 and 43% respectively. *Methanobacteriales* were the third most abundant group ranging between 6 and 15% in relative abundance. 1–5% of the observed sequences were annotated as *Methanomassiliicoccales*, a *Thermoplasmatales* related methanogenic archaeal order (Paul et al., 2012; Sollinger et al., 2016). The overall community composition was consistent with the findings based on the metagenomic 16S rRNA gene sequences. Here, sequences classified as *Methanosarcinales* were the most dominant group with 50% followed by *Methanomicrobiales* with 43%. Sequences classified as *Methanobacteriales* and *Methanomassiliicoccales* each accounted for 4% of the community.

The sequences from the bacterial domain were dominated by the classes *Clostridia* (23–42%), *Bacteroidia* (10–23%), OPB54 belonging to the *Firmicutes* (12–19%), and *Mollicutes* (6–21%), accounting together for over 67% of all sequences in the primer-based datasets. An exception to this was the uncultured “*Candidatus*” *Cloacimonetes* of the SHA-4 candidate division belonging to the *Spirochaetes*. Sequences of these organisms were almost exclusively detected with primer pair BacV35 where it accounted for 17% of all sequences.

Concerning the 16S rRNA gene sequences extracted from the metagenome, the most prominent observation was the higher abundance of the “*Candidatus*” *Cloacimonas* sequences with 16% and the lower abundance of the *Clostridia* with only 18% compared to the primer based method. *Bacteroidia* (16%) and *Mollicutes* (2%) were among the highly abundant taxa as well. Additionally, 16S rRNA gene sequences with no classification below the domain level were of higher abundance compared to the amplicon based approaches. The proportion of sequences originating from Archaea was 2.4% while 97.6% of the sequences were classified as Bacteria.

### Alpha diversity analysis differed highly between tested primer pairs

**Figure 5:**
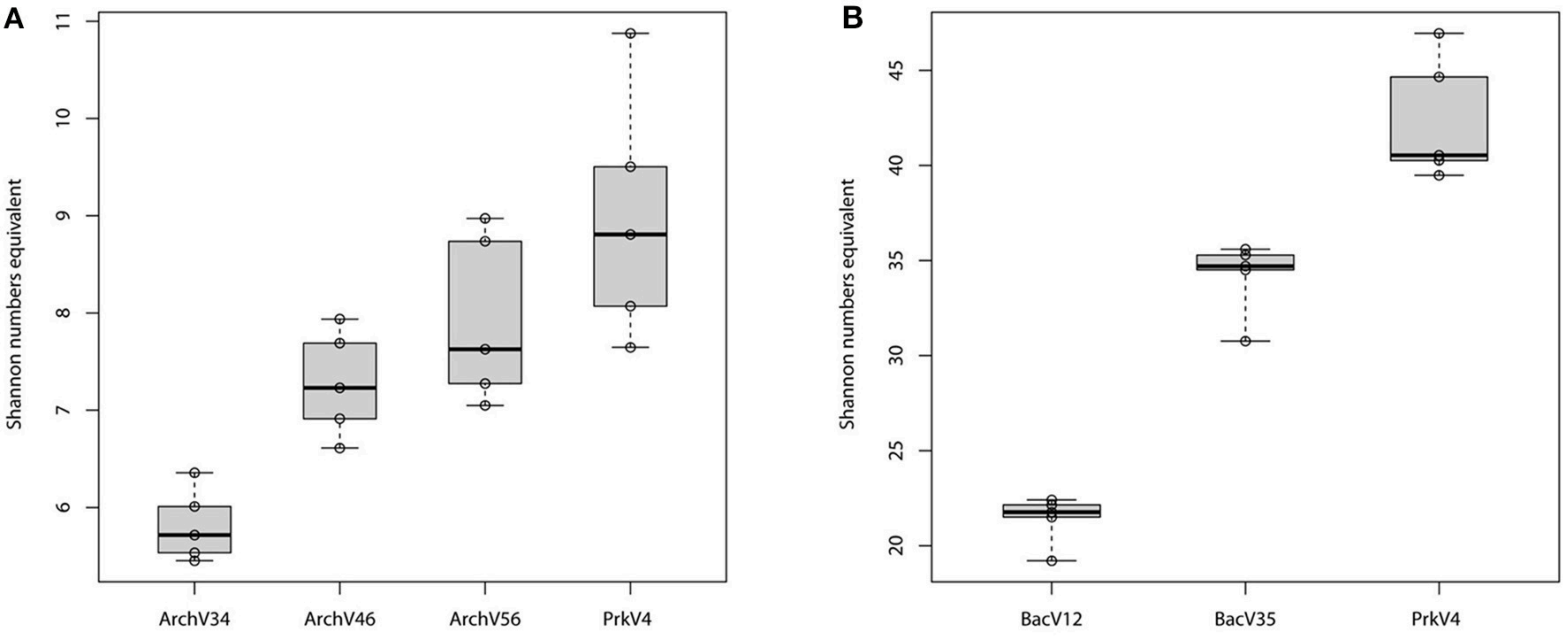
**Alpha diversity (Shannon numbers equivalent) observed on the 99% OTU level in the tested archaeal (A) and bacterial (B) datasets amplified with the primer pairs (***n*** = 5) after removal singletons and low abundant OTUs (below 0.2%)**.

Alpha diversity was analyzed for the different datasets based on the OTU composition generated at the 99% sequence similarity level (see Figure 5) as well as for the 97% sequence similarity level (see Figure S2), both summarized in Table 3. The observed diversity depends on (i) the potential of the amplified region to discriminate between different taxonomic units, (ii) the similarity cutoff value for OTU separation and (iii) the range of species amplified by the primer pair. On the 99% similarity level, the datasets of the primer pairs ArchV34 showed lower median alpha diversity of 5.7 for the archaeal domain, diversity was comparably high based on primer pair ArchV46, ArchV56 and PrkV4 with 7.2, 7.6, and 8.8. In contrast, alpha diversity was overall higher within the bacterial datasets. Bacterial diversity observed with primer pair PrkV4 was particularly high with 40.5, while the primer pairs BacV12 and BacV35 resulted in Shannon numbers equivalent of 21.8 and 34.7, respectively.

**Table 3:** **Summary of the potential of the tested primer pairs ***in-silico*** and in application**.

**Primer set**		**Specificity [%]**	**Alpha diversity**			**Beta diversity**
		**Experimental**	**Database coverage observed [%]**	**Observed** α**-Diversity in the dataset**		**Dissimilarity to metagenome**
			**OTU 97%**		**OTU 99%**	
	ArchaeaV34	10.9	74.0	2.4	5.7	0.145
	ArchaeaV46	16.4	79.0	4.1	7.2	0.290
	ArchaeaV56	64.5	76.5	2.7	7.6	0.197
PrkV4	Archaea	3.2	88.6	4.9	8.8	0.213
	Bacteria	96.8	88.2	41.2	40.5	0.489
	BacteriaV12	100.0	60.2	23.9	21.8	0.453
	BacteriaV35	99.9	85.6	32.5	34.7	0.462

*Specificity refers to the percentage of sequences belonging to the targeted domain in the final dataset used for the analysis. Alpha diversity includes the potential coverage as calculated by the use of the SILVA-database version 123 and the observed alpha diversity calculated for the experimental datasets. Beta diversity summarizes the mean Bray-Curtis dissimilarity between the primer pairs and the 16S rRNA gene sequence composition extracted from the shotgun metagenome*.

### Beta diversity measurement

**Figure 6:**
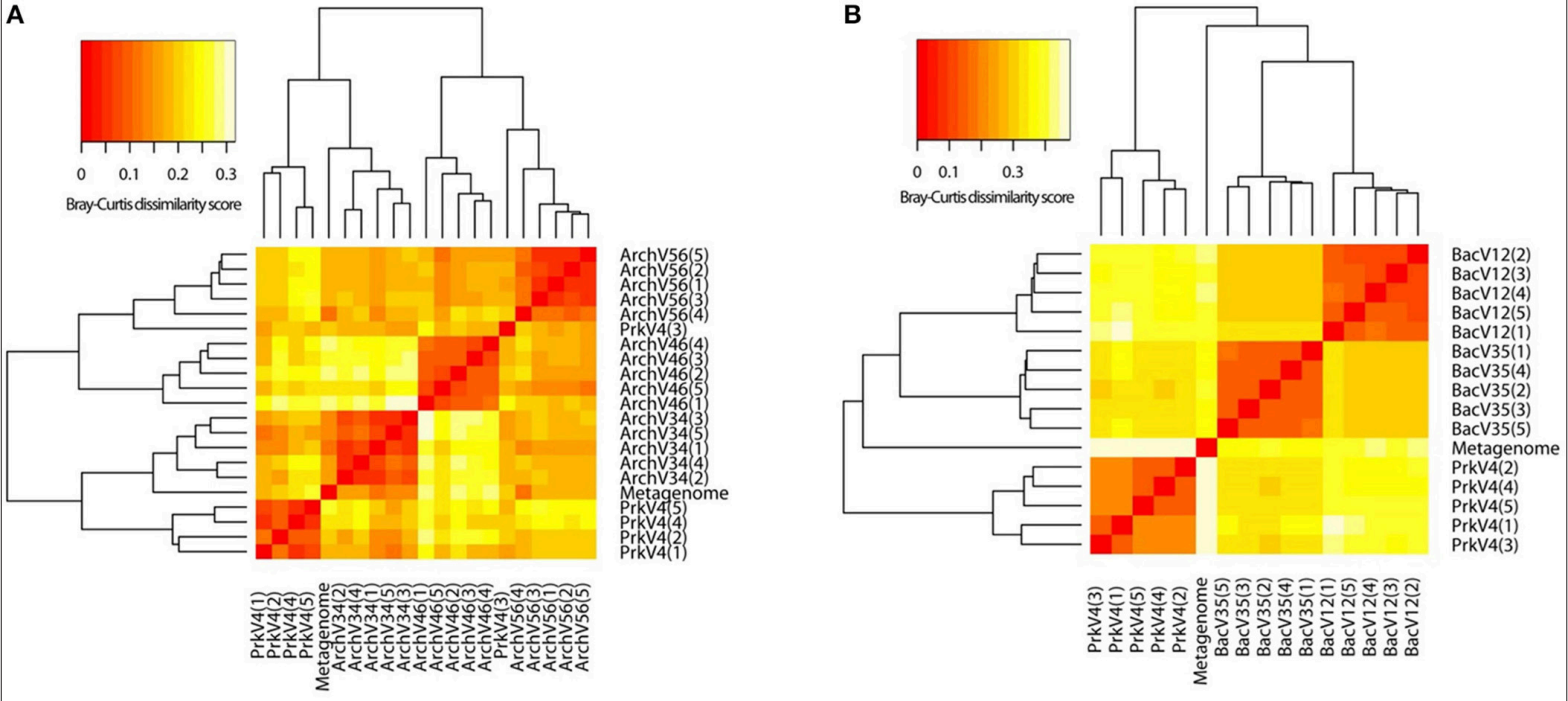
**Heat map derived from dissimilarity matrix of Bray-Curtis distances between archaeal (A) and bacterial (B) community compositions as observed by the primer pairs and the extracted metagenomic 16S rRNA gene sequences**. Similarity between samples is indicated as a color gradient from higher similarity (red) to lower similarity in (white).

For comparison of variation in the observed archaeal and bacterial sequence composition, beta diversity was analyzed applying redundancy analysis (RDA) based on abundances of taxonomic bins at the genus level. Therefore, the merged tax.summary files holding information on the abundance of different genera in the communities observed by the primer pairs were transformed to a taxon-per-site table. This taxonomy based approach was chosen since comparison at the usual OTU level was impossible between the different primer pairs. After Hellinger transformation of count data, a Bray-Curtis dissimilarity matrix was calculated. The results are shown as a heat map in Figure 6. Higher similarity is indicated by red colors whereas low similarity between observed communities is indicated by white. Clustering of the replicates in the archaeal primer pairs (Figure 6A) was observed for the ArchV34, ArchV34, and ArchV56 as well as for four of five replicates of the PrkV4 primer pairs. In the bacterial dataset, the clustering of the replicates was stronger compared to the archaeal dataset (Figure 6B).

**Figure 7:**
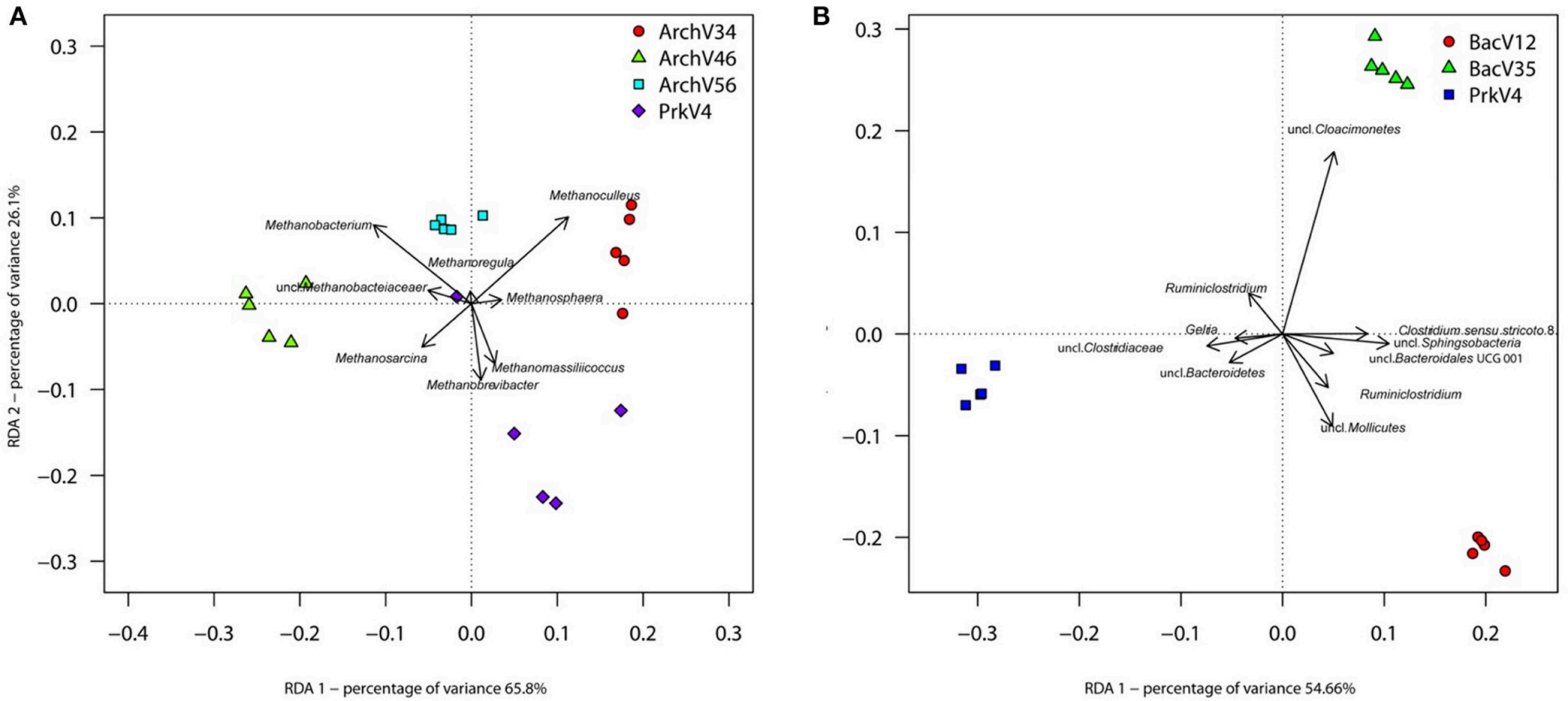
**RDA based on Hellinger-transformed taxon count data after Bray-Curtis dissimilarity calculation**. Clear separation of the different primer pairs can be observed between the different archaeal **(A)** and bacterial **(B)** communities observed in the sample. The eight (Archaea) and 10 (Bacteria) taxa contributing most of the variance of the dataset are shown as vectors.

Redundancy analysis based on the Hellinger transformed data was performed using the command *rda* in R with “primer pairs” as explanatory factor. Overall adjusted variances explained by the first two RDA dimensions as shown in Figure 7 were 69.35% for the archaeal dataset (Figure 7A) and 83.53% for the bacterial dataset (Figure 7B). Distinctly separated clusters of the different datasets indicate a specific image of the community for each primer pair. The model was tested for significance by an analysis of variance (ANOVA) using the command *anova* with 1000 permutations. Results showed high significance for the archaeal [*F*_(3, 16)_ = 15.336; *p* = 0.001] and bacterial [*F*_(2, 12)_ = 36.509; *p* = 0.001] models. Upon significance of the complete model, pairwise comparisons were conducted between the respective archaeal and bacterial primer pair datasets. Benjamini-Hochberg procedure was applied to correct *p*-values to account for pairwise testing (Benjamini and Hochberg, 1995). *F*-values ranged from 53.791 to 6.4614 with corresponding *q*-values between 0.0132 and 0.0260 for archaeal data (summarized in Table S2). *F*-values for pairwise testing of bacterial primer pairs ranged from 52.012 to 29.381 with corresponding *q*-values from 0.0135 to 0.0140 (summarized in Table S3).

To further investigate differences between observed community structures, indicator taxa were determined as previously described by Weiland-Bräuer et al. (2015) using the command *multipatt* of the R package indicspecies v.1.7.1 (Cáceres and Legendre, 2009) with 10^5^ permutations. Indicator taxa are characteristic for a certain environment, or—as in our case—for a primer pair. Therefore, the analysis of indicator species may indicate possible over- or underestimation of certain taxa by the tested primer pairs, which is an important criterion for primer choice and reliability. Six indicator taxa from the archaeal domain were found to be characteristic in the datasets for the different primer pairs (summarized in Table S4). Sequences classified as *Methanosphaera* and *Methanoculleus* were found to be characteristic for the communities observed by primer pair ArchV34, whereas communities amplified by primer pair ArchV46 were more characterized by the presence of sequences annotated as *Methanobacterium* and *Methanosarcina*. The prokaryotic primer pair PrkV4 was characterized by *Methanobrevibacter* and *Methanomassiliicoccus* sequences. No particularly characteristic taxon was found in the dataset generated with the ArchV56 primer pair.

For the bacterial domain, indicator taxon analysis found 10 taxa to be characteristic for the different primer pairs (summarized in Table S5). BacV12 was characterized by sequences annotated as uncultured WCHB1 69 belonging to the *Sphingobacteriales, Ruminiclostridium*, as well as uncultured taxa annotated as *Mollicutes, Clostridia* and *Bacteroidales*. Sequences annotated as uncultured *Cloacimonetes* were characteristic for primer pair BacV35. Primer pair PrkV4 had *Ruminiclostridium, Gelria* as well as two uncharacterized sequences annotated as *Clostridiaceae* and *Bacteroidetes* found to be characteristic for the dataset. Interestingly, the observed indicator taxa did not correlate with higher *in-silico* database coverage of a primer pair for that specific taxon and can therefore not be predicted based on the database analysis. The above mentioned indicator taxa contributed most to the variance explained by RDA and are visualized as vectors in Figure 7.

## Discussion

The choice of the primer pair for 16S rRNA gene amplification substantially determines quality and perspective on the obtained community data. Numerous recent studies address the topic of comparability between 16S rRNA gene based projects, demonstrating the influences of different effects with the help of mock communities and simulated datasets (Schloss et al., 2011; Brooks et al., 2015; Tremblay et al., 2015). However, complexity of environmental samples cannot be fully mimicked by artificially generated communities and the effects due to the choice of the primer pairs for analyzing complex environmental samples remain in question. Whereas most of the above mentioned studies focus mainly on Bacteria, here we presented comprehensive data generated for the archaeal and bacterial fraction of a complex environment, where we observed similar tendencies of primer effects in both domains. Based on our results five core statements can be formulated:

- All primer pairs were able to recover and represent a typical complex microbial community of an anaerobic biogas reactor, yet with a different outcome concerning the details of community structure. Sequences of key organisms for major steps in hydrolysis, acidogenesis and acetogenesis mostly belonging to the *Clostridia, Bacteroidia*, and *Actinobacteria* were observed in all bacterial datasets. The archaeal datasets provided sequences of species capable of hydrogenotrophic, acetoclastic, and methylotrophic methanogenesis (Wirth et al., 2012; Sundberg et al., 2013). Especially in the sequences of the archaeal community, a clear abundances ranking of taxonomic orders (*Methanosarcinales* > *Methanomicrobiales* > *Methanobacteriales* ≥ *Methanomassiliicoccales*) was consistently conserved in all tested primer pairs as well as in the sequences obtained from the metagenome. Similarly, ranking order of the two most abundant bacterial classes (*Clostridia*> *Bacteroidia*) was conserved in the sequence abundance in all bacterial datasets. In the metagenome derived 16S rRNA gene sequences, the classes *Clostridia* and *Bacteroidia* were highly abundant (18 and 16%) but in addition sequences of unclassified *Cloacimonetes* contributed 16% to the dataset (Figure 4). This taxon would have been missed using the primer pairs BacV12 or PrkV4 in the analysis of the environment. Organisms of this class were recently found in metagenomic datasets from anaerobic digesters (Solli et al., 2014) and are expected to participate in syntrophic degradation of fatty acids and protein intermediates (Pelletier et al., 2008; Limam et al., 2014). The lower proportion of *Clostridia* in the metagenomic 16S rRNA gene sequences might indicate an overestimation of this class in the amplicon based approaches. In our case, the most promising combination to analyze the community of the sample would have been the combination of the BacV35 and ArchV46 primer pairs, however the metagenomic sequences still show a different overall bacterial and archaeal community compared to that observed by those primer pairs (Figure 6).
- From a technical point of view, efficiency strongly differed between the evaluated primer pairs, namely due to unspecific amplification by archaeal primer pairs. While 65% of the raw reads from the primer pair ArchV56 could be used for the analysis, the read reduction for the primer pair ArchV34 left only 11% of the raw reads for the final analysis of the archaeal community. Reads filtered from the archaeal datasets mostly belonged to the bacterial domain, with high abundance of the class “*Candidatus*” *Cloacimonas*. While almost all removed sequences from the primer pair ArchV34 belonged to this class, sequences removed from the datasets of the primers ArchV46 and ArchV56 showed a higher diversity but, as expected, were not comparable in composition to the tested bacterial primer pairs (Figure S1). Low specificity of the ArchV34 primer pair toward archaea was not predicted by the Silva TestPrime tool (Klindworth et al., 2013), the reason for this being unclear. One of the primers applied in this primer pair, S-D-Arch-0787-a-A-20, showed potential unspecific amplification within the bacterial domain in the *in-silico* prediction. Inexplicably, the same primer was used as reverse complement in the primer pair ArchV56, which exhibited highest specificity of all archaeal primer pairs tested. This shows that the outcome of sequencing runs is still highly unpredictable and database results cannot be directly transferred to the wet lab application.
- Alpha diversity (Shannon numbers equivalent ^1^D) differed between primer pairs. In general, higher observed alpha diversity for a specific primer pair indicates higher resolution of the present diversity (i.e., better separation of OTUs) in a given sample. The alpha diversity in general as well as the Shannon number equivalents was lower in the archaeal datasets compared to the bacterial. This observation has previously been made for comparable biogas reactors (Francisci et al., 2015). The Shannon numbers equivalent was selected as alpha diversity metric as it is more robust in the application in environmental data and can be seen as the number of equally abundant species needed to form the diversity observed within a given dataset (Jost et al., 2010). For the primers targeting the archaeal 16S rRNA gene regions, the observed alpha diversity correlates well with the prediction of the database coverage (see Table 3). High diversities were observed for the primer pairs ArchV46 (^1^D = 7.23) and PrkV4 (^1^D = 8.81) covering the highly variable region V4 (Cai et al., 2013). In combination with a high potential coverage of different phyla, ArchV46 thus appears to be quite promising and was recently applied for the analysis of the archaeal domain in a mesophilic anaerobic digesters (Goux et al., 2015). The high diversity observed in the PrkV4 dataset correlates well with the high theoretical coverage as predicted by the *in-silico* evaluation. In our study, the abundances of the domains Archaea and Bacteria were similar in the overall metagenomic (2.4–97.6%) and the amplicon based (3.2–96.8%) 16S rRNA gene sequences generated by primer pair PrkV4, thus showing no strong shift in the proportions of the two domains during amplicon generation. The ArchV56 dataset showed diversity within the same range, a finding which correlated well with the *in-silico* predicted coverage. The observed positive correlation of *in-silico* database coverage and observed diversity was also valid for the tested bacterial primer pairs.
- Beta diversity analysis showed good reproducibility within a primer pair, but poor comparability between primer pairs as shown in the heat map (Figure 6). Significant differences between the communities amplified by the tested primer pairs resulted from differential amplification of 16S rRNA gene sequences out of the same starting material as well as the ability of the amplified variable region to discriminate between different taxa (Shakya et al., 2013; Tremblay et al., 2015). This bias in amplification and classification is also assumed to be the reason for the observed differences compared to the metagenome derived sequences. The comparison to 16S rRNA gene sequences from metagenomic data has previously shown to conform with 16S rRNA amplicon sequences, generated from environmental samples like sheep rumen (Shi et al., 2014). For the archaeal dataset generated by the PrkV4, we observed lower clustering in the RDA (Figure 7A) as well as higher within group variance, which could already be observed in the community composition (Figure 3). This resulted from overall lower sequencing depth of the archaeal domain in this dataset. As mentioned before, the overall archaeal abundance within the dataset was 3.2% compared to the also targeted bacterial sequences. As described by Kittelmann et al. (2013), the application of multiple domain specific primer sets can be beneficial when strong differences in the abundance of the different domains can be observed in a habitat. In the before mentioned study, the rumen communities of several ruminants were investigated by the application for several specific primer sets targeting Bacteria, Archaea, Protozoa and Fungi and pooled in alternating proportions for sequencing to account for the different abundances of the rumen microbiota (Kittelmann et al., 2013). In conclusion, to reduce the within group variation of the PrkV4 archaeal dataset an increased number of sequences would be needed for a saturating analysis of the archaeal domain using the general PrkV4 primer pair. As alternative, a separate analysis of the archaeal and bacterial domain might be beneficial for the investigated habitat.
- To give an advice on which primer pair to use is difficult, if not impossible since the choice depends on the habitat and the research question. However we summarized the results obtained in this study in Table 3 to better compare the dis-advantages of the primer pairs tested in the presented study. Primer pair ArchV34 showed the highest similarity to the archaeal domain of the metagenomic results. Still, it cannot be fully recommended since it was highly unspecific for archaeal sequences. Due to this fact, only 11% of the sequences obtained for this primer pair could be used for analysis. The ArchV46 primer pair showed moderate specificity and high diversity which makes it a reliable candidate for the investigation of new archaeal taxa in diverse environments. It was successfully applied in a recent multi-omics approach investigating the archaeal domain in an anaerobic paddy field. In this complex environment it was able to detect a complex archaeal community, consisting mostly *of Methanomicrobia, Methanomassiliicoccales and Methanobacteria*, as well as some *Thermoprotei* (Ogawa et al., 2014). The primer pair ArchV56 showed the highest specificity for archaeal 16S rRNA gene sequences. Compared to the metagenomic archaeal community, the observed diversity and similarity was average, which makes this primer pair a decent choice for the detection of archaea, especially in environments with a low abundance of archaea. In combination with a fluorescent probe, this primer pair was originally designed for the quantification of archaea (Yu et al., 2005) and was applied in this context in diverse studies (Lee et al., 2008; Nettmann et al., 2008). Beside good theoretical coverage and high diversity, a clear advantage of the PrkV4 primer pair is the simultaneous amplification of archaea and bacterial 16S rRNA gene sequences which can be helpful in the investigation of synergies between the archaeal and bacterial domain in the environment. This advantage was already confirmed in other environmental studies like in the investigation of the coral-associated microbiota containing substantial amounts of *Thaumarchaeota* and minor amounts of *Euryarchaeota*, which were detected in coral mucus for the first time (van Bleijswijk et al., 2015). Unfortunately, compared to the bacterial 16S rRNA gene sequences extracted from the metagenome, the sequences generated with the PrkV4 primer pair showed the lowest similarity. One prominent prokaryotic primer pair is applied in the earth microbiome project (Caporaso et al., 2012). The PrkV4 primer pair showed higher *in-silico* coverage compared to the one applied in the earth microbiome project, still this study cannot provide a direct comparison. The primer pair BacV12 has been used in diverse medical and environmental studies so far (Rausch et al., 2011; Cozen et al., 2013; Langfeldt et al., 2014; Mensch et al., 2016). Even though the primer pair sowed the highest similarity to the bacterial domain observed in the metagenome, the low theoretical coverage and low observed diversity within the samples may hint toward a possible non-observance of present species. Average results in terms of diversity and similarity to the metagenomic results were observed for the primer pair BacV35. The amplification of the highly variable region 4 of the 16S rRNA gene qualifies this primer pair as a good candidate when the focus of the study lies on the bacterial domain only (Güllert et al., 2016).

In summary, it appears most beneficial to use the same primer pair when comparing different sites or environments by amplicon sequencing. This assumption has previously been made for bacterial communities (Baker et al., 2003; Frank et al., 2008; Tremblay et al., 2015) and, as shown here, is also valid for archaeal primer pairs. It should be mentioned that additional effects influencing the observed community structure also occur in form of nucleic acid extraction (LaMontagne et al., 2002; Brooks et al., 2015), the kits applied (Adams et al., 2015), PCR artifacts (Schloss et al., 2011; Brooks et al., 2015), and database bias (Werner et al., 2012), as well as bias introduced by selected hypervariable region (Chakravorty et al., 2007; Yu et al., 2008), the sequencing platform itself (Kim et al., 2011; Luo et al., 2012; Tremblay et al., 2015) or the sequencing center (Hiergeist et al., 2016). In addition, comparability between communities analyzed with different primer pairs is bound to taxonomically assigned sequences and therefore limited and biased by the completeness of the database (Werner et al., 2012). These aspects further emphasize the need for general standards when planning and conducting environmental microbiological research for the sake of improved comparability, like the human (Turnbaugh et al., 2007; Peterson et al., 2009) or earth microbiome project guidelines (Gilbert et al., 2010) with profound and detailed manual and instruction for the sample preparation, which is of great help for between-study comparability. Finally, a comparison to 16S rRNA gene sequences gained from a corresponding metagenome as presented here appears very helpful and can be recommended as an addition to the mock community testing (Brooks et al., 2015; Tremblay et al., 2015) for the evaluation of new archaeal or bacterial primer pairs, especially when the community composition of the investigated environment is yet undetermined.

## Author contributions

MF designed the study, carried out experiments, analyzed, and interpreted the data, and wrote the manuscript. SG provided metagenomic data and designed the study. SN supported the statistical analyses on the sequencing data. WS provided metagenomic data and designed the study. RS designed the study, founded, and supervised the work. All authors gave input to the manuscript and participated in the writing process.

## Funding

Financial support was provided by the BMBF project BioPara funded to RS (grant no. 03SF0421B) and WS (grant no. 03SF0421H). We acknowledge financial support by Land Schleswig-Holstein within the funding program Open Access Publikationsfonds.

### Conflict of interest statement

The authors declare that the research was conducted in the absence of any commercial or financial relationships that could be construed as a potential conflict of interest.
