# Supplementary material for: Evaluation of 16S rRNA Gene Primer Pairs for Monitoring Microbial Community Structures Showed High Reproducibility within and Low Comparability between Datasets Generated with Multiple Archaeal and Bacterial Primer Pairs
Source: Front Microbiol. 2016 Aug 23;7:1297. doi: 10.3389/fmicb.2016.01297 (PMC4994424; doi:10.3389/fmicb.2016.01297)
Supplement: Supplementary file 1 [file Table1.DOCX]

Supplementary Material

# Evaluation of 16S rRNA gene primer pairs for monitoring archaeal and bacterial community structures: A comparative study estimating method-based biases for archaeal primer pairs

M. A. Fischer^1^, S. Güllert^2^, S. C. Neulinger^1,3^, W. R. Streit^2^, R. A. Schmitz^1^*

*** Correspondence:** R. A. Schmitz: rschmitz@ifam.uni-kiel.de

Table S 1: Number of sequences during the mothur pipeline steps. The numbers in brackets list the unique sequences in the dataset. The number of OTUs per dataset was calculated on the 97% cut-off level.

| **Pair name** | **start** | **screen.seqs & filter.seqs** | **chimera.perseus** | **get.lineage** | **OTUs** |
| --- | --- | --- | --- | --- | --- |
| **ArchV56** | 48803 (10772) | 43425 (8441) | 41538 (5835) | 26371 (2175) | 177 |
| **ArchV46** | 35874 (24643) | 32655 (14778) | 25933 (7886) | 4306 (354) | 90 |
| **ArchV34** | 52587 (6732) | 51425 (5923) | 51104 (2504) | 5763 (934) | 75 |
| **BacV35** | 37183 (24562) | 35236 (13081) | 29672 (6462) | 29604 (6408) | 2144 |
| **BacV12** | 73733 (22025) | 58679 (16701) | 53371 (6666) | 48317 (5521) | 2702 |
| **PrkV4 (Archaea)** | 70038 (18803) | 44408 (12389) | 41888 (4390) | 1294 (151) | 55 |
| **PrkV4 (Bacteria)** |  |  |  | 40023 (4061) | 1467 |
