# Supplementary material for: Evaluation of 16S rRNA Gene Primer Pairs for Monitoring Microbial Community Structures Showed High Reproducibility within and Low Comparability between Datasets Generated with Multiple Archaeal and Bacterial Primer Pairs
Source: Front Microbiol. 2016 Aug 23;7:1297. doi: 10.3389/fmicb.2016.01297 (PMC4994424; doi:10.3389/fmicb.2016.01297)
Supplement: Supplementary file 3 [file Table3.DOCX]

Supplementary Material

# Evaluation of 16S rRNA gene primer pairs for monitoring archaeal and bacterial community structures: A comparative study estimating method-based biases for archaeal primer pairs

M. A. Fischer^1^, S. Güllert^2^, S. C. Neulinger^1,3^, W. R. Streit^2^, R. A. Schmitz^1^*

*** Correspondence:** R. A. Schmitz: rschmitz@ifam.uni-kiel.de

Table S 3: F-values and q-values (gray) of the pairwise comparison for the bacterial sequence composition generated with the tested primer pairs. q-values were calculated using Benjamini Hochberg correction.

|  | BactV12 | BactV35 | PrkV4 |
| --- | --- | --- | --- |
| BactV12 |  | 0.0135 | 0.0140 |
| BactV35 | 52.012 |  | 0.0135 |
| PrkV4 | 35.647 | 29.381 |  |
