# Supplementary material for: Evaluation of 16S rRNA Gene Primer Pairs for Monitoring Microbial Community Structures Showed High Reproducibility within and Low Comparability between Datasets Generated with Multiple Archaeal and Bacterial Primer Pairs
Source: Front Microbiol. 2016 Aug 23;7:1297. doi: 10.3389/fmicb.2016.01297 (PMC4994424; doi:10.3389/fmicb.2016.01297)
Supplement: Supplementary file 4 [file Table4.DOCX]

Supplementary Material

# Evaluation of 16S rRNA gene primer pairs for monitoring archaeal and bacterial community structures: A comparative study estimating method-based biases for archaeal primer pairs

M. A. Fischer^1^, S. Güllert^2^, S. C. Neulinger^1,3^, W. R. Streit^2^, R. A. Schmitz^1^*

*** Correspondence:** R. A. Schmitz: rschmitz@ifam.uni-kiel.de

Table S 4: Results for the archaeal indicator species analysis. The first rows state the primer set a Taxon is indicator for.

| Taxon | ArchV34 | ArchV46 | ArchV56 | PrkV4 | index | stat | p-value | q-value |
| --- | --- | --- | --- | --- | --- | --- | --- | --- |
| Methanosphaera | 1 | 0 | 0 | 0 | 1 | 0.6962 | 0.0005 | 0.001 |
| Methanoculleus | 1 | 0 | 0 | 0 | 1 | 0.6618 | 0.0006 | 0.001 |
| Methanobacterium | 0 | 1 | 0 | 0 | 2 | 0.6493 | 0.0007 | 0.001 |
| Methanosarcina | 0 | 1 | 0 | 0 | 2 | 0.6231 | 0.0091 | 0.009 |
| Methanobrevibacter | 0 | 0 | 0 | 1 | 4 | 0.6935 | 0.0004 | 0.001 |
| Methanomassiliicoccus | 0 | 0 | 0 | 1 | 4 | 0.6402 | 0.0039 | 0.005 |
