# Supplementary material for: Evaluation of 16S rRNA Gene Primer Pairs for Monitoring Microbial Community Structures Showed High Reproducibility within and Low Comparability between Datasets Generated with Multiple Archaeal and Bacterial Primer Pairs
Source: Front Microbiol. 2016 Aug 23;7:1297. doi: 10.3389/fmicb.2016.01297 (PMC4994424; doi:10.3389/fmicb.2016.01297)
Supplement: Supplementary file 5 [file Table5.DOCX]

Supplementary Material

# Evaluation of 16S rRNA gene primer pairs for monitoring archaeal and bacterial community structures: A comparative study estimating method-based biases for archaeal primer pairs

M. A. Fischer^1^, S. Güllert^2^, S. C. Neulinger^1,3^, W. R. Streit^2^, R. A. Schmitz^1^*

*** Correspondence:** R. A. Schmitz: rschmitz@ifam.uni-kiel.de

Table S 5: Results for the bacterial indicator species analysis. The first rows state the primer set a Taxon is indicator for.

| Species | BacV12 | BacV35 | PrkV4 | index | stat | p-value | q-value |
| --- | --- | --- | --- | --- | --- | --- | --- |
| uncl.Bacteroidales UCG 001 | 1 | 0 | 0 | 1 | 0.7247 | 0.0007 | 0.002 |
| uncl.WCHB1 69 | 1 | 0 | 0 | 1 | 0.7605 | 0.0012 | 0.002 |
| Clostridium sensu stricto 8 | 1 | 0 | 0 | 1 | 0.7384 | 0.0008 | 0.002 |
| Ruminiclostridium (1) | 1 | 0 | 0 | 1 | 0.9751 | 0.0014 | 0.002 |
| uncl.Mollicutes | 1 | 0 | 0 | 1 | 0.8365 | 0.001 | 0.002 |
| uncl.Cloacimonetes | 0 | 1 | 0 | 2 | 0.9574 | 0.0007 | 0.002 |
| uncl.Bacteroidetes | 0 | 0 | 1 | 3 | 0.8527 | 0.0012 | 0.002 |
| uncl.Clostridiaceae | 0 | 0 | 1 | 3 | 0.9415 | 0.0013 | 0.002 |
| Ruminiclostridium (2) | 0 | 0 | 1 | 3 | 0.6969 | 0.0483 | 0.048 |
| Gelria | 0 | 0 | 1 | 3 | 0.8881 | 0.0024 | 0.003 |
