# Supplementary material for: Evaluation of 16S rRNA Gene Primer Pairs for Monitoring Microbial Community Structures Showed High Reproducibility within and Low Comparability between Datasets Generated with Multiple Archaeal and Bacterial Primer Pairs
Source: Front Microbiol. 2016 Aug 23;7:1297. doi: 10.3389/fmicb.2016.01297 (PMC4994424; doi:10.3389/fmicb.2016.01297)
Supplement: Supplementary file 6 [file Image1.PDF]

Supplementary Material

Evaluation of 16S rRNA gene primer pairs for monitoring archaeal and bacterial community structures: A comparative study estimating method-based biases for archaeal primer pairs

M. A. Fischer[1], S. Güllert[2], S. C. Neulinger[1,3], W. R. Streit[2], R. A. Schmitz[1]*

*** Correspondence:** R. A. Schmitz: rschmitz@ifam.uni-kiel.de

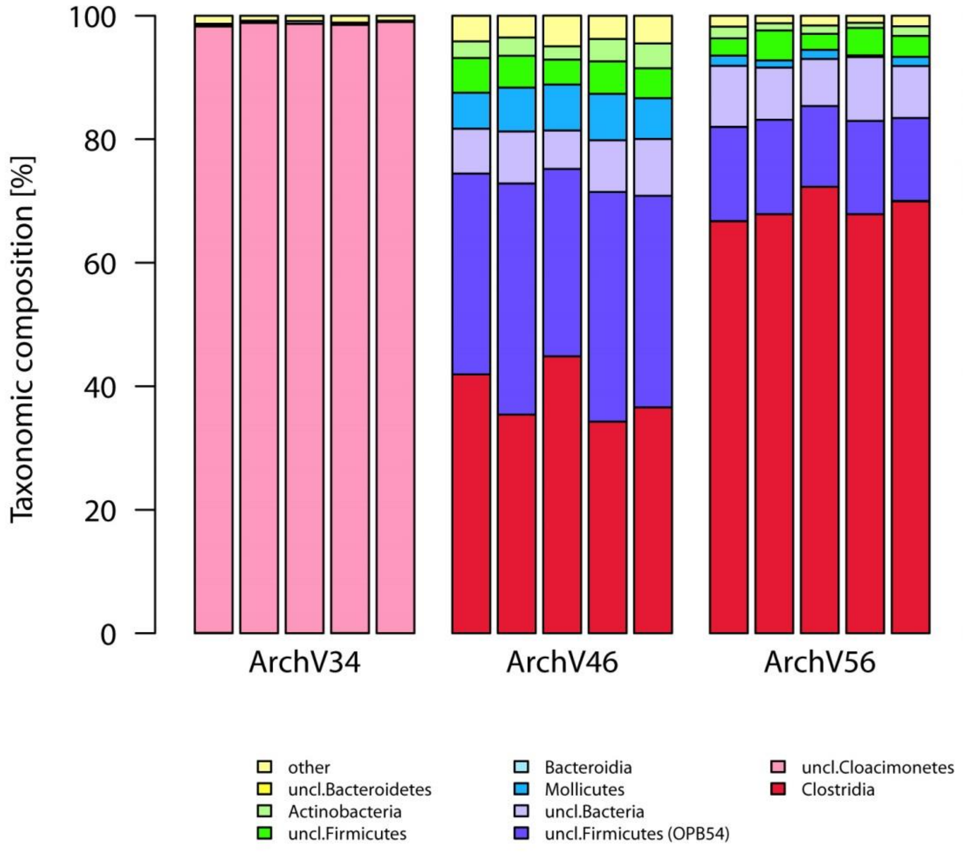

Supplementary Figure 1. : Taxonomic annotation based on the SILVA v123 database of the bacterial 16S rRNA gene Sequence co-amplified within the datasets generated with the primers designed for the detection of archaeal 16S rRNA gene sequences.
