# Supplementary material for: Evaluation of 16S rRNA Gene Primer Pairs for Monitoring Microbial Community Structures Showed High Reproducibility within and Low Comparability between Datasets Generated with Multiple Archaeal and Bacterial Primer Pairs
Source: Front Microbiol. 2016 Aug 23;7:1297. doi: 10.3389/fmicb.2016.01297 (PMC4994424; doi:10.3389/fmicb.2016.01297)
Supplement: Supplementary file 7 [file Image2.PDF]

*Supplementary Material*

# Evaluation of 16S rRNA gene primer pairs for monitoring archaeal and bacterial community structures: A comparative study estimating method-based biases for archaeal primer pairs

**M. A. Fischer[1], S. Güllert[2], S. C. Neulinger[1,3], W. R. Streit[2], R. A. Schmitz[1]***

*** Correspondence:** R. A. Schmitz: rschmitz@ifam.uni-kiel.de

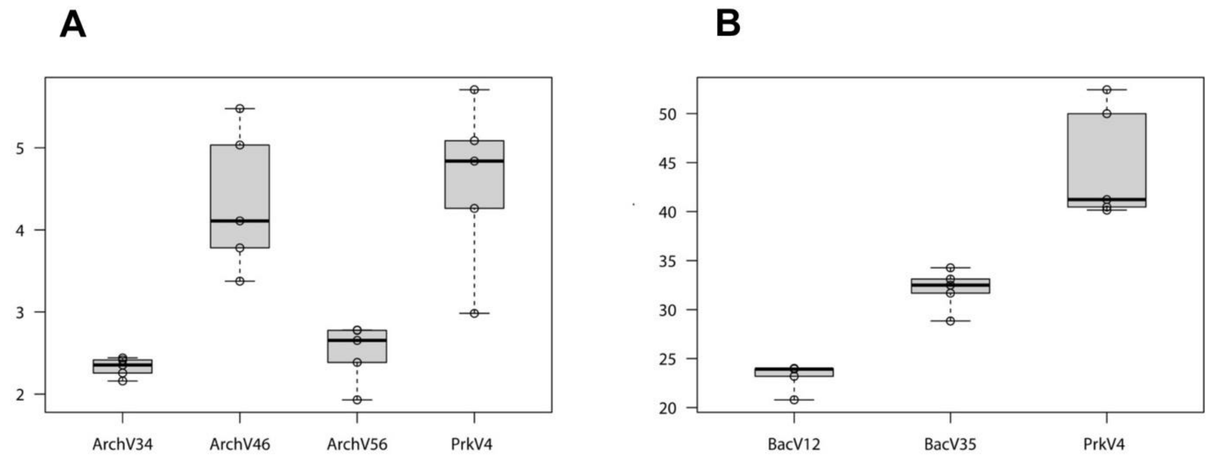


**Supplementary Figure 2.** : Alpha diversity (Shannon numbers equivalent) observed on the 97 % OTU level in the tested archaeal (A) and bacterial (B) datasets amplified with the primer pairs (n=5) after removal singletons and low abundant OTUs (below 0.2%).
